# Prediction and Prediction Error in Autism: A Meta-Analysis of Functional Magnetic Resonance Imaging Results

**DOI:** 10.1016/j.bpsgos.2026.100760

**Published:** 2026-05-15

**Authors:** Hjalmar Nobel Norrman, Yating Huang, Annelies van’t Westeinde, Tessa M. van Leeuwen, Peter Fransson, Sven Bölte, Janina Neufeld

**Affiliations:** a Center of Neurodevelopmental Disorders at Karolinska Institutet, Centre for Psychiatry Research, Department of Women’s and Children’s Health, Karolinska Institutet, Stockholm Health Care Services, Stockholm, Sweden; b Pediatric Endocrinology Unit, Department of Women’s and Children’s Health, Karolinska Institutet, Karolinska University Hospital, Stockholm, Sweden; c Pediatric Endocrinology Unit, Department of Clinical Sciences, Sahlgrenska Academy, University of Gothenburg, Sahlgrenska University Hospital, Gothenburg, Sweden; d Department of Communication and Cognition, Tilburg School of Humanities and Digital Sciences, Tilburg University, Tilburg, the Netherlands; e Donders Institute for Brain, Cognition and Behaviour, Radboud University, Nijmegen, the Netherlands; f Department of Clinical Neuroscience, Karolinska Institutet, Stockholm, Sweden; g Curtin Autism Research Group, Curtin School of Allied Health, Curtin University, Perth, Western Australia, Australia; h Child and Adolescent Psychiatry, Stockholm Health Care Services, Stockholm, Sweden; i Swedish Collegium for Advanced Study, Uppsala, Sweden

**Keywords:** Activation likelihood estimation (ALE), Autism, Functional magnetic resonance imaging (fMRI), Meta-analysis, Predictive processes, Review

## Abstract

**Background:**

In autism spectrum condition (ASC), altered priors/predictions or prediction errors have been hypothesized to increase the influence of bottom-up sensory input, relative to top-down prior knowledge. Such alterations could account for several autistic features, but their empirical basis is unclear. In neurotypical (NT) individuals, multiple neuroimaging meta-analyses have aimed to outline domain-general prediction networks of the brain. However, there has not been a similar meta-analysis in autism.

**Methods:**

We performed a literature search for functional magnetic resonance imaging and magnetoencephalography studies with autistic participants. The contrasts that explicitly or implicitly involved the processing of priors/predictions and prediction errors were selected. Contrasts were divided into those that reported stronger activation in ASC compared with NT groups (ASC > NT; 8 contrasts; 139 ASC, 150 NT) and vice versa (NT > ASC; 13 contrasts; 261 ASC, 289 NT). The convergence of differences between the groups was then tested using activation likelihood estimation meta-analysis. We also identified 37 contrasts without significant group differences.

**Results:**

ASC > NT did not result in significant convergence. NT > ASC converged in a cluster in the medial frontal gyrus/cingulate gyrus.

**Conclusions:**

We found converging NT > ASC activation differences in an area associated with error monitoring and uncertainty estimation. Our results are generally consistent with notions of altered predictions or prediction errors in autism, pointing to differences at high levels of the information-processing hierarchy. However, we recommend a cautious interpretation, given the limited number of available contrasts and the high proportion of null results.

## Plain Language Summary

Several features of autism have been hypothesized to be related to alterations in the way the brain predicts upcoming events and inputs. In this meta-analysis of fMRI studies, we show that when autistic and neurotypical groups engage in tasks and activities that directly or indirectly involve predictions, converging brain activation differences are observed in the anterior cingulate cortex, a region involved in uncertainty estimation and error detection. While this finding could be consistent with hypotheses of prediction-related differences in autism, we also find that over half of fMRI experiments do not detect reliable group differences.

Autism spectrum condition (ASC) is defined by a diverse set of behavioral features in both social and nonsocial domains, including insistence on sameness and sensory hypersensitivity (1). Bayesian and predictive coding accounts of autism propose that altered priors/predictions or prediction errors increase the influence of bottom-up sensory inputs, relative to top-down prior beliefs (2). This imbalance hypothesis (2) could, if correct, potentially account for several autistic features in both social and nonsocial domains (3,4).

One hypothesis of autistic perception is of broader Bayesian priors (5). In Bayesian perception models, the cause of noisy sensory input is inferred by multiplying the likelihood of the sensory input given each potential cause with the prior probability of potential causes. The resulting posterior distribution of probabilities of causes, given the sensory input, could then inform perceptual decision (6, 7, 8). In autism, broader priors would shift the posterior closer to the mean of sensory inputs (9), resulting in a, metaphorically, more literal perception. Besides broader priors, it has been pointed out that supposing a decreased variance of likelihoods—that is, less noisy sensory inputs—would have the same effect (9).

Related accounts of autism are based on predictive coding (3,4,10), according to which perception is generated by a hierarchical model distributed along the cerebral cortex. Information generated from sensory inputs flows forward in the hierarchy, and model predictions flow backward. The interpretation of sensory inputs is constrained by predictions (i.e., priors, from hereon used interchangeably). When the sensory input is not explained by predictions, the prediction error is used to update them (11). The influence of prediction errors in revising predictions depends on their estimated reliability (their inverse variance, or precision) (12). One hypothesis is that autism is characterized by a uniformly high precision assigned to prediction errors. Precise errors would increase the updating of predictions, causing some to be overfitted to the sensory input (4).

Through different mechanisms, these hypotheses of autism suppose an increased influence of bottom-up sensory input, relative to top-down processing (2). However, the empirical basis for such an imbalance is unclear. A recent review of autism/autistic traits, covering behavioral, neuroimaging, and eye tracking evidence, found a tendency toward impairment in the development of new priors. Overall though, less than half of the reviewed findings supported an imbalance (2). Another review (13), framed in terms of a related proposal (14), which also included a mix of (partly overlapping, partly different) studies, found that the evidence overall suggested differences in prediction-related learning and low-level prediction errors (13). Both studies decided against a meta-analysis, in part due to the variety of the reviewed evidence (2,13). The lack of empirical clarity underlines the need to further investigate altered predictions in autism, including the neural processes supposed to underlie them.

In neurotypical (NT) participants, neural processes associated with predictions have been studied extensively. Functional magnetic resonance imaging (fMRI) studies have found activation consistent with mismatch error effects in the auditory cortex (15) and with the encoding of Bayesian surprise in the anterior cingulate cortex (ACC) (16). A decreased neural response to predictable stimuli has been interpreted as sensory input being accounted for by feedback signals, and therefore more efficiently encoded (17). Others have separated top-down/bottom-up signaling (18) and distinguished between expected/unexpected events (19) across layers of the early visual cortex, consistent with an idea of spatial separability between predictions and prediction errors (18,19). Meanwhile, in autism, prediction-related alterations have been hypothesized in the ACC (3,4,14), insula (4), basal ganglia (14), and cerebellum (14). A specific example of an hypothesis would be increased activation in the visual cortex during object perception, as bottom-up input would be less modulated by top-down predictions compared with NT participants (20).

In NT participants, fMRI results have been aggregated in several meta-analyses, some aiming to identify domain-general prediction networks of the typical brain (21, 22, 23). These meta-analyses gathered fMRI experiments^a^ reflecting 2 separate processes: first, the formation, encoding, or reinforcement of predictions, and second, the violation of predictions.^b^ When analyzing these 2 processes together, results differed, but also converged in approximately similar areas in the right superior frontal gyrus (22,23), the left (21, 22, 23)/right (21,23) inferior frontal gyrus, the left (21, 22, 23)/right (21,23) (anterior) insula, the left superior temporal sulcus/gyrus (21,23), and the left precuneus (22,23). Note however that because Costa *et al.* (23) included all experiments in the meta-analyses by Siman-Tov *et al.* (21) and Ficco *et al.* (22), results between them are partially dependent.

Meta-analytic results outlining prediction networks in NT participants could inform the investigation of autism, as differences in identified regions could lend indirect support to the imbalance hypothesis. However, there has not been a meta-analysis of autism corresponding to those in NT participants. In our study, we used a wide-ranging literature search to identify fMRI and magnetoencephalography (MEG) studies with autistic participants. From these studies, we selected experiments that explicitly or implicitly involved the processing of predictions/prediction errors. Then, we evaluated the convergence of between-group activation differences (ASC > NT, NT > ASC) in 2 activation likelihood estimation (ALE) analyses.

## Methods and Materials

This meta-analysis was registered in PROSPERO (24) in February 2024 (CRD42024503295). We followed the Preferred Reporting Items for Systematic Reviews and Meta-Analyses (PRISMA) recommendations (25) and recommendations for neuroimaging meta-analyses (26).

### Literature Search

The 2 previous reviews of evidence relating to predictions in autism (2,13) each included fewer than 10 studies using fMRI or MEG. Meanwhile, the authors observed that they did not necessarily capture all relevant studies, since similar paradigms can be framed in different terms (2,13). We instead used a terminology-neutral search to capture any conceptually relevant study, irrespective of framing.

**Figure 1:**
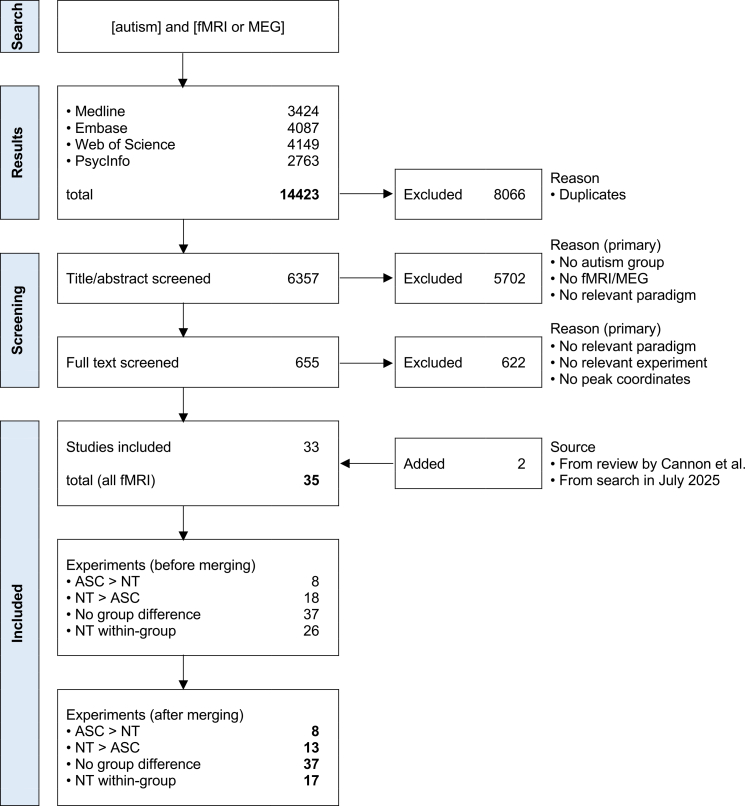
Overview of the literature search, study selection, and experiment extraction. Adapted from the Preferred Reporting Items for Systematic Reviews and Meta-Analyses (PRISMA) 2020 flow diagram (https://www.prisma-statement.org/prisma-2020-flow-diagram) (25). ASC, autism spectrum condition; NT, neurotypical; fMRI, functional magnetic resonance imaging; MEG, magnetoencephalography.

We used variations on [autism] and [fMRI or MEG] and searched in MEDLINE, Embase, Web of Science, and PsycInfo for studies published between 1990 and December 2023 (Tables S1–S4). The search resulted in 6357 unique studies (Figure 1). One study (27) referenced in the review by Cannon *et al.* (13) was added. Another study was added after a renewed search in July 2025 (28). Screening of reference lists in included studies did not lead to further additions.

### Selection

**Figure 2:**
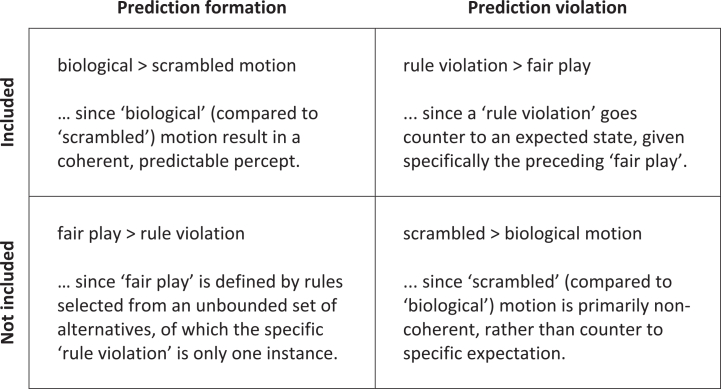
Examples of categorization of experiments that provide sufficient (included) and insufficient (not included) contrast for prediction formation and violation.

To achieve comparability, the overall selection, classification, and analysis strategies were modeled approximately on those of prior ALEs in NT participants (21, 22, 23). Studies were assessed on the following criteria: 1) English language, peer-reviewed, and original study; 2) ASC and NT group: regardless of age and type of autism diagnosis, though not associated with a genetic syndrome; 3) fMRI or MEG; 4) a paradigm that explicitly or implicitly involves the formation or violation of predictions, irrespective of whether these were already existing or acquired within the paradigm, of sensory modality, or whether there was an active task; 5) whole-brain, between-group experiment of 2 active conditions, or a parametric modulator, providing sufficient contrast for either prediction formation or violation (Figure 2); and 6) Montreal Neurological Institute (MNI) or Talairach coordinates of peak activation.

Criteria 1 to 4 were assessed by 2 authors during blind, independent title/abstract screening. Decisions were intermittently unblinded to assess agreement. Criteria 5 and 6 were assessed by one author during full-text screening. Decisions on the inclusion or exclusion of paradigms and experiments were regularly discussed in a wider author group.

### Paradigm Classification

In this meta-analysis, prediction formation covered paradigms that involved the forming of expectation or—more implicitly—that involved top-down influence on perception, like in object or speech recognition. Such paradigms were included if the comparator condition consisted of similar visual/auditory elements as the object condition (controlling for general sensory features) and was sufficiently scrambled or nonfigurative (controlling for top-down influence). A conceptually related type of task fulfilling these criteria was single-word–nonword paradigms. However, we argue that this type of paradigm does not provide sufficient contrast, and therefore excluded it.

Prediction violation covered paradigms where expectation was subverted. Congruency-incongruency type paradigms were included if the number of congruent trials was at least equal to that of incongruent trials to avoid confounding between frequency and semantic content. Paradigms where incongruent responses could be explained by communicative intent (e.g., irony) were excluded, as context can render those responses pragmatically nonsurprising.

Mismatch negativity effects have been interpreted in terms of both adaptation (29) and predictive coding (30). We included auditory and visual oddball experiments, as their structure (deviant > standard) was assumed to provide sufficient contrast for prediction violation (criterion 5) (Figure 2) under a predictive coding interpretation. Meanwhile, we excluded repetition suppression experiments, since their structure (early > late) was assumed to provide insufficient contrast for prediction formation under criterion 5, regardless of interpretation.

### Extraction

Paradigm specifics, between-group experiments, coordinates of peak activation, statistical threshold, group sizes, gender, age, and NT within-group experiments were extracted by one author. For studies with significant between-group differences, behavioral group differences relevant for the included fMRI experiments and quality metrics were also extracted. Coordinates, group sizes, and quality metrics were checked by a second author.

Guided by previous reports of mixed evidence (2), besides the positive evidence, we also extracted between-group experiments without significant differences (null results). A null result was defined as no difference at the most conservative between-group threshold. This definition included, for example, analyses of variance (ANOVAs) without significant interaction effects or ANOVAs without group differences in the between-condition direction identified as relevant for this meta-analysis (criterion 5) (Figure 2).

Where available, we additionally extracted significant NT within-group experiments. Previous ALEs in NT participants used differing selection criteria (21, 22, 23), and our criteria differed individually from those. For example, we included oddball and learning paradigms [in contrast to Siman-Tov *et al.* (21)], excluded repetition suppression paradigms [in contrast to Ficco *et al.* (22)], and excluded experiments of the type standard > deviant sound [in contrast to Costa *et al.* (23)]. By comparing our NT results with previous ALEs, we could assess to what extent we captured the same neural processes, informing the interpretation of between-group results.

Where applicable, we inquired with the authors of studies for information on sample sizes/dependencies and for whole-brain (between-group) results, if they were not reported. We also inquired in cases where paradigms hypothetically permitted specific prediction formation/violation (between-group) experiments. As a result, experiments from one study (28) were added.

### Analysis

ALE analysis evaluates the spatial convergence across a set of peak activation coordinates (foci) against a null distribution, with the number of individuals (observations) determining the spatial uncertainty of each focus (31, 32, 33). Foci from dependent experiments—that is, from the same participant group—were merged if contributing to the same analysis (26,33).

We performed the ALE analysis in GingerALE version 3.02 (https://www.brainmap.org/ale/), using the smaller (conservative) gray matter mask, the recommended thresholding: voxel-level *p* < .001, cluster-level *p* < .05 (26), familywise error (FWE) corrected (26,34), and using 1000 permutations. Talairach coordinates were converted to MNI152 in GingerALE with the icbm2tal and mni2tal transforms. Results were visualized in MRIcroGL (https://www.nitrc.org/projects/mricrogl/) on the mni152 standard template.

As null results cannot be represented in an ALE (35), we have reported them descriptively. To complement this description, we assessed the robustness of the ALE results by an adapted fail-safe N (FSN) analysis (35) (https://github.com/NeuroStat/GenerateNull). Here, null results are represented by experiments with a random foci distribution. The proportion of null experiments that can be added before clusters of significant convergence turn nonsignificant is tested. If this proportion is lower than the estimated real proportion of null results, it could indicate a risk for a nonrobust ALE result (35).

## Results

We identified 35 fMRI studies with relevant between-group experiments. There were no eligible MEG studies, primarily due to a lack of reported peak activation coordinates. Nineteen studies reported at least one between-group experiment with significant differences (Table 1; see Table S5 for sample demographics and quality metrics).

**Table 1:** Between-Group Experiments Included in the Meta-Analysis

Author	Group Size^a^	Paradigm	Experiment	Prediction
ASC > NT				

Bolling *et al.* (73)	21 ASC, 19 NT	Coplayers follow or break the rules of a game	Rule violation > fair play (Cybershape)	Violation
Gomot *et al.* (74)	12 ASC, 12 NT	Repetitions of standard, novel, and deviant sounds (task version)	Novel > standard	Violation
Groen *et al.* (75)	16 ASC, 26 NT	Normal spoken sentences or speech-like noise fragments	Normal sentence > speech-like noise	Formation
Hames *et al.* (76)	6 ASC, 6 NT	Flankers congruent or incongruent with target	Incongruent > congruent	Violation
Kinard *et al.* (39)	22 ASC, 20 NT	Cues probabilistically signal upcoming reward or nonreward	Thresholded unsigned prediction error (social condition)	Violation
Libero *et al.* (40)	21 ASC, 22 NT	Agent uses an object in an ordinary or unusual way	Unusual > ordinary	Violation
Sapey-Triomphe *et al.* (20)	16 ASC, 19 NT	Elements move to form recognizable or nonrecognizable objects	Meaningful > meaningless (contour and texture)	Formation
Sapey-Triomphe *et al.* (69)	25 ASC, 26 NT	Alternating tones signal upcoming (ambiguous or unambiguous) rotation direction	Precision-weighted prediction error (second level)	Violation

NT > ASC				
Balsters *et al.* (27)	16 ASC, 20 NT	Cues signal upcoming reward or nonreward for various agents	Prediction error > predicted outcome (all agents)	Violation
Björnsdotter *et al.* (77)	37 ASC, 37 NT^b^	Elements represent human motion or scrambled motion	Coherent > scrambled motion (replication cohort)	Formation
Bolling *et al.* (73)	21 ASC, 19 NT	Coplayers follow or break the rules of a game	Rule violation > fair play (Cybershape)	Violation
Caria *et al.* (78)	8 ASC, 14 NT	Music or random sequence of tones	(Happy) music > control (standard and favorite)	Formation
			(Sad) music > control (standard and favorite)	Formation
D’Cruz *et al.* (37)	17 ASC, 23 NT	Set-shifting task	First reversal > first and following expected (four-choice task)	Violation
Fan *et al.* (38)	12 ASC, 12 NT	Cue signal location of an upcoming target	Invalid > valid cue	Violation
		Flankers congruent or incongruent with target	Incongruent > congruent	Violation
Freitag *et al.* (79)	14 ASC, 14 NT	Elements represent human motion, or scrambled motion	Biological > scrambled motion	Formation
Gomot *et al.* (80)	12 ASC, 12 NT	Repetitions of standard, novel, and deviant sounds (nontask version)	Deviant > standard	Violation
			Novel > standard	Violation
Gomot *et al.* (74)	12 ASC, 12 NT	Repetitions of standard, novel, and deviant sounds (task version)	Novel > standard	Violation
Groen *et al.* (75)	16 ASC, 26 NT	Last word of a statement does or does not violate real-world knowledge	Anomaly > no anomaly (world knowledge)	Violation
Jack *et al.* (48)	45 ASC, 45 NT	Elements represent human motion, or scrambled motion	Biological > scrambled motion (matched female sample)	Formation
Shafritz *et al.* (41)	15 ASC, 14 NT	Repetitions of standard, novel, and target shapes	Target > (5 last) standard	Violation
			Novel > (5 last) standard	Violation
Sharer *et al.* (81)	17 ASC, 36 NT	Target locations follow a set or a random sequence	Sequence > random (run 1)	Formation
Yang *et al.* (82)	31 ASC, 17 NT	Elements represent human motion, or scrambled motion	Biological > scrambled (full sample)	Formation

ASC, autism spectrum condition; NT, neurotypical.

a Participants after exclusions and/or within the relevant subset, to the extent specified.

b Information here assumes a typo in the referenced study from which group 1 participant was excluded to achieve equal sample sizes.

### ALE: ASC > NT Experiments

Eight experiments reported ASC > NT activation differences (37 foci; 139 ASC, 150 NT). The ALE analysis did not reveal any statistically significant clusters of convergence.

### ALE: NT > ASC Experiments

**Figure 3:**
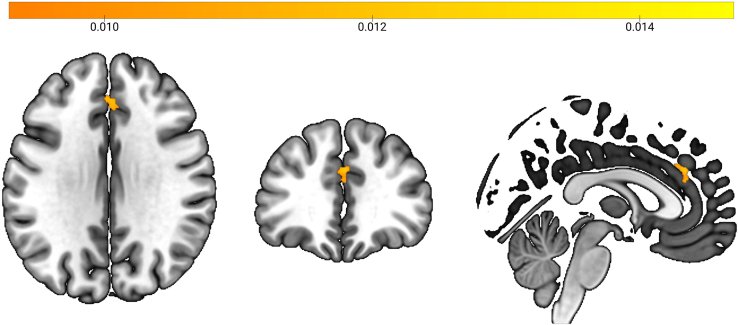
Converging neurotypical > autism spectrum condition activation differences in the medial frontal gyrus/cingulate gyrus. Slice displayed, x = 0.93, y = 34.51, z = 30.00.

Eighteen experiments reported NT > ASC activation differences (142 foci; 261 ASC, 289 NT). Merging dependent experiments reduced this number to 13. Activation differences converged in a single, 2-peak 744-mm^3^ cluster in the medial frontal gyrus (MeFG), in Brodmann area (BA) 9, and the cingulate gyrus (CG) (BA 32) (Figure 3 and Table 2). The cluster intersected BA 24 and BA 32, overlapping the dorsal parts of ACC (dACC) (36). Three experiments (27,37,38) reflecting prediction violation contributed foci to the cluster.

**Table 2:** Neurotypical > Autism Spectrum Condition Results

Area	Volume, mm^3^	ALE	*z*	*p*	MNI		
					x	y	z
Medial Frontal Gyrus	744	0.012	3.76	<.0005	−2	36	28
Cingulate Gyrus	–	0.011	3.49	<.0005	0	36	22

ALE, activation likelihood estimation; MNI, Montreal Neurological Institute.

### Group Differences on Behavioral Measures

Of the 19 studies with between-group activation differences, 12 included one or more behavioral measures relevant for this meta-analysis (Table S6). Seven studies, including 2 that contributed foci to the significant NT > ASC cluster, reported significant group differences on at least one measure. Generally, these results showed a pattern of higher accuracy rates in NT. Accuracy was higher in reporting whether an outcome was expected/unexpected (27), a reward/nonreward was likely (39), an action was ordinary/unusual (40), an infrequent target shape was presented (41), and in reporting the direction of incongruently, relative to congruently, presented targets (38).

### Null Results and FSN Analysis

Across the 35 included studies, there were 37 null experiments (Table S7), equal to a total proportion of 58.73%. The share of prediction formation/violation was similar between significant (34.61%/65.38%) and nonsignificant (35.13%/64.86%) experiments. Discounting studies with dependent samples, mean group sizes were also relatively similar in those with (19.50 ASC, 21.27 NT) and without (21.68 ASC, 22.06 NT) (28,42, 43, 44, 45, 46, 47, 48, 49, 50, 51, 52, 53, 54, 55, 56, 57) differences. However, there appeared to be differences between the 2 sets of studies in statistical thresholding. Of 18 studies with significant group differences, 5 used a threshold other, and presumably more lenient, than corrected for multiple comparisons at a cluster-level *p* ≤ .05, compared with none of 16 studies without significant group differences.

In the FSN analysis, random NT > ASC experiments proportionate to 50% were added to the ALE. The MeFG/CG cluster did not remain significant, indicative of low robustness.

### ALE: NT Within-Group Experiments

We found 26 NT within-group experiments (17 after merging) (277 foci; 395 NT) (Table S8). There were 2 resulting clusters (Figure S1 and Table S9)—one with peaks in the left supramarginal gyrus and the inferior parietal lobule [approximately similar to Costa *et al.* (23)] and one in the left claustrum [approximately similar to Siman-Tov *et al.* (21), Ficco *et al.* (22), and Costa *et al.* (23)], indicating at least partial overlap with previously captured processes.

## Discussion

In this meta-analysis, we aggregated fMRI results that explicitly or implicitly relate to the processing of predictions/prediction errors. NT > ASC group activation differences converged in a cluster in the MeFG/CG, overlapping dACC. The location of differences is interesting from a theoretical perspective, as dACC has been proposed to have a domain-general role in tracking goal-related information such as error, conflict, and surprise (58). This information is integrated to determine the expected value of control, for example, in regulating attentional resources. This monitoring and control function explains why dACC is active during a wide range of paradigms and particularly under new and complex conditions which require nonautomatic control (58).

There are some parallels to this functional description of dACC in predictive coding perspectives on the role of attention (59). Herein, attentional gain has been interpreted as a situational increase in the weighting of task-relevant prediction errors (60). The influence of prediction errors should increase when unexpected information motivates an update of task-relevant predictions. Whether an outcome is considered unexpected or not—that is, whether it represents changing contingencies—depends on higher-order estimates of expected uncertainty. In predictive coding accounts of autism, altered volatility/uncertainty modeling has been a key process of interest, in which ACC specifically is theorized to be involved (3,4,61,62).

The structure of experiments contributing foci to the dACC cluster implies that in NT participants, there was a comparatively stronger response to outcomes that conflict with expectation, previous cues, or learning history. Or stated differently, that under these conditions, dACC activity in autism differentiated less between unexpected and expected events. This pattern would conform empirically to some previous results in autism from probabilistic learning. There, behavioral and pupillometric measures indicated a reduced distinction between expected/unexpected outcomes, coupled with increased estimates of higher-order volatility (61)—though see Angeletos Chrysaitis and Seriès (2) for a note on the reproducibility of the latter in related, but nonidentical, studies.

Against this background, converging differences in (d)ACC could be interpreted as generally consistent with proposals of altered predictions/prediction errors in autism. However, we note that activation differences alone have limited power to identify an underlying mechanism. To illustrate, the NT > ASC result could be interpreted to reflect uniformly precise prediction errors in autism, regardless of whether outcomes could be considered expected or not. But the same pattern could also be interpreted to support broader Bayesian priors, to the extent that violations of weaker priors would generate comparatively weaker prediction errors. These interpretations rest on opposite assumptions of the strength of baseline error signaling, while activation differences itself could be consistent with either.

Previous ALEs in NT participants have identified other subregions of both MeFG and CG as being parts of a general prediction network (23), though not consistently (21,22). One explanation for the lack of consistency could be that ACCs’ general function (e.g., its role in error detection) makes it specifically sensitive to prediction violation, relative to formation, experiments—convergence therefore potentially being suppressed when the 2 types of experiments are analyzed jointly. This reasoning is supported by ACC consistently being identified in other meta-analyses focused exclusively on prediction error (63, 64, 65, 66). However, note that in previous meta-analyses, contrast/separate analysis of prediction violation specifically did not necessarily identify ACC either (21,22).

There was no significant ASC > NT convergence, possibly due to lack of power. One interpretation of the lower number of ASC > NT experiments is that autistic individuals were overall less sensitive to the contrasts between conditions. While, again, the underlying mechanism cannot be inferred, such a pattern would be generally consistent with a reduced differentiation between expected/unexpected outcomes that these and others’ results point to. Another, less specific factor could be a previously observed increase in variability in brain activity (67)/connectivity (68) between autistic individuals, decreasing the overall probability of significant effects in ASC over NT participants.

Guided by prior reports of mixed evidence for a predictive imbalance in autism (2), we also described the counterevidence to group differences in brain activation. The share of null results, approximately three-fifths, seems to complement the trend in prior work (2)—though a precise comparison is made difficult by differences in the definition of relevant data. At least from the selection of experiments in this meta-analysis, it appears that the fMRI evidence for generally altered predictions/prediction errors in autism is inconsistent. While sample sizes were similar between studies with and without significant findings, we cannot exclude that differences in statistical thresholding could have contributed to separating them. In addition, robustness analysis showed that the observed proportion of null findings warrants a careful interpretation of our ALE results. Overall, this picture could be indicative of a previously stated need to develop more specific hypotheses (2,13).

A subset of included studies used behavioral measures that could, for our purposes, strengthen confidence in the theoretical relevance of activation results. However, many of the studies leveraged in this meta-analysis were not designed to investigate predictions/prediction errors specifically. To allow for additional validation and inference on mechanisms, activation results would optimally be supplemented by a combination of behavioral measures and, as previously emphasized (2), appropriate computational modeling. To take one example, Sapey-Triomphe *et al.* (69) modeled the influence of a hierarchy of priors and prediction errors on task behavior and then correlated it with brain activation results.

We agree with the authors of previous reviews in hoping that the field will continue to develop (2,13) and that increasing numbers of specialized studies will allow for a more refined meta-analysis in the future. Relative to many of the rather indirect measures of predictions/prediction errors leveraged here, such studies could potentially have more power to detect relevant differences. If so, it is possible that they could yield fewer null results.

### Limitations

The main limitation of this meta-analysis is the small number of included experiments. First, it incurs a risk that a single or a few influential experiments drive results. Simulation work on ALE analysis using FWE correction indicates that at least 17 to 20 experiments should be included per analysis to mitigate this risk (34). After merging dependent experiments, the maximum number we reached in any between-group analysis was 13. The limited amount of positive evidence restricts our ability to draw strong conclusions from the results, which should be replicated with a larger sample size.

Second, a small sample size restricts its power to detect true differences. This might be especially relevant for differences associated with prediction formation. Prediction violation experiments are conceptually similar, across sensory modalities and tasks, in that they reflect the subverting of expectation. Prediction formation experiments, on the other hand, share a looser definition and here involved processes such as object recognition, pattern recognition, and the general intelligibility of stimuli. If brain activation associated with these different processes is more variable, like noted in prior work (22,23), then underrepresentation of specific tasks or sensory modalities could limit potential convergence.

For related reasons, our results, in showing convergence of differences at high levels of the information-processing hierarchy, do not provide strong evidence against differences in areas such as the sensory cortices. The domain-independent scope of our analysis should inherently bias convergence specifically to areas with integrative functions, corresponding to high levels (11) of the generative model in the predictive coding framework. However, by assumption, the model is distributed at all levels of the brain. For example, if autistic prediction error signaling would be stronger in the sensory cortices, our analysis would be unlikely to identify it.

Subanalyses would have been the main instrument both to control for variability and to further investigate the results. Given more data, a natural step would have been to analyze prediction formation and violation experiments separately. Separate analysis would have been warranted, particularly given that different hypotheses of autism emphasize on the one hand altered priors/predictions and on the other hand prediction errors. Another relevant analysis would have been to compare acquired and existing predictions, given indications that evidence could be relatively stronger for differences in the development of new priors (2).

Finally, a related limitation is the heterogeneity of participant age groups in the included studies. Including all ages increases not only power but also variability. In autism, there are age-dependent patterns of resting-state connectivity differences (70), which conceivably could affect task-related brain activation (71,72). It is therefore possible that diverging differences between age cohorts could have censored age-dependent between-group effects.

In summary, our inferences are possibly more reliable for prediction violation–related differences and likely limited spatially to brain areas with domain-independent function. Because subanalyses were unfeasible, we could not investigate whether different kinds of predictions are differentially altered in autism or exclude that different sources of variability suppressed true convergence of differences.

### Conclusions

We have presented an ALE meta-analysis of fMRI results that explicitly or implicitly relate to the processing of predictions/prediction errors in autism. Our results show converging NT > ASC activation differences in ACC, a region associated with error monitoring and uncertainty estimation. The structure of contributing experiments indicates that this effect could have been driven by a reduced differentiation between expected and unexpected outcomes in autism, at least under some conditions. Such a reduction would be consistent with hypotheses of altered predictions/prediction errors generally [see for example, Lawson *et al.* (61)], although we cannot infer by what mechanism.

Meanwhile, concerns over generalizability are raised by the small number of available experiments—increasing the risk of chance convergence—and, similar to that previously observed (2), a high proportion of null results. For a conclusive and detailed assessment, additional neuroimaging studies specifically targeting predictions/prediction errors in autism are needed. Optimally, these studies should include behavioral measures and computational modeling to inform the interpretation of brain activation results.

## Supplementary Material

Figure S1 and Tables S1–S9

Key Resources Table

## Footnotes

a A note on terminology used in the present meta-analysis: Study refers to an individual paper, paradigm to a task/stimuli used in a study, and experiment refers to a contrast of brain activation between conditions in a paradigm.

b Siman-Tov *et al*. (21) included paradigms from three separate domains (including action perception, music, and language), while Ficco *et al*. (22) and Costa *et al*. (23) used broader selections. Experiments were defined as reflecting prediction formation/violation (21), encoding/violation (22), and congruency/incongruency (23).
