# Supplementary material for: Prediction and Prediction Error in Autism: A Meta-Analysis of Functional Magnetic Resonance Imaging Results
Source: Biol Psychiatry Glob Open Sci. 2026 May 15;6(5):100760. doi: 10.1016/j.bpsgos.2026.100760 (PMC13319389; doi:10.1016/j.bpsgos.2026.100760)
Supplement: Figure S1 and Tables S1–S9 [file mmc1.pdf]

SUPPLEMENTARY INFORMATION

Prediction and Prediction Error in Autism: A Meta-Analysis of Functional Magnetic Resonance Imaging Results

Nobel Norrman *et al.*

SUPPLEMENTARY INFORMATION

Prediction and prediction error in autism: A meta-analysis of functional magnetic resonance imaging results

Supplementary Tables 3

Table S1. Search Terms: Medline 3

Table S2. Search Terms: Embase 4

Table S3. Search Terms: Web of Science 5

Table S4. Search Terms: PsycInfo 6

Table S5. Demographic and Quality Metrics 8

Table S6. Group Differences on Behavioral Measures 10

Table S7. Non-Significant Between-Group Experiments 12

Table S8. NT Within-Group Experiments 15

Table S9. NT Within-Group Results 19

Supplementary Figures 19

Figure S1. NT Within-Group Results 19

References 19

Supplementary Tables

Table S1. Search Terms: Medline

No	Terms	Results
1	exp Child Development Disorders, Pervasive/	48 965
2	(Autism* or autist* or asperger* or Kanner* Syndrome*).ti,ab,kf.	69 586
3	(pervasive adj3 (development* or disorder*)).ti,ab,kf.	2 776
4	or/1-3	74 470
5	Functional neuroimaging/	4 773
6	Brain Mapping/	99 292
7	Echo-Planar Imaging/	4 963
8	Magnetic Resonance Imaging/ and functional*.ti,ab,kf.	76 428
9	Magnetoencephalography/	9 408
10	((echoplanar* or echo planar* or functional) adj3 (neuroimag* or image or images or imaging* or scan or scans or mri or tomograph*)).ti,ab,kf.	81 288
11	(functional cerebral localization* or fMRI or magnetoencephalogra* or magneto-encephalogra* or "magneto encephalogra*" or MEG?).ti,ab,kf.	80 557
12	(brain* adj1 (activ* or connectiv* or correlate* or function* or image or images or imaging* or mapping* or network* or resonance* or respons* or tomograph* or radiograph*)).ti,ab,kf.	119 366
13	or/5-12	285 197
14	4 and 13	4 697
15	exp In Vitro Techniques/	624 493
16	in vitro.ti,ab,kf.	1 455 719
17	15 or 16	1 896 506
18	14 not 17	4 662
19	18 not (animals not humans).sh.	4 449
20	limit 19 to (english language and yr="1990 -Current")	4 325
21	limit 20 to (clinical conference or congress or consensus development conference or consensus development conference, nih)	1
22	20 not 21	4 324
23	(review or systematic review or meta analysis).pt. or (review or meta analysis or metaanalysis).ti.	3 635 762
24	22 and 23	900
25	22 not 23	3424

Interface: Ovid MEDLINE(R) ALL.

Table S2. Search Terms: Embase

No	Terms	Results
#01	'autism'/exp	100 932
#02	autism*:ti,ab,kw OR autist*:ti,ab,kw OR asperger*:ti,ab,kw OR 'kanner* syndrome*':ti,ab,kw	90 523
#03	(pervasive NEAR/3 (development* OR disorder*)):ti,ab,kw	4176
#04	#1 OR #2 OR #3	113 374
#05	'functional magnetic resonance imaging'/de	103 346
#06	'brain mapping'/de	37 885
#07	'brain radiography'/de	9 885
#08	'magnetoencephalography'/de	14 774
#09	'brain tomography'/de	3 250
#10	'echo planar imaging'/de	8 481
#11	'nuclear magnetic resonance imaging'/de AND functional*:ti,ab,kw	81 274
#12	((echoplanar* OR 'echo planar*' OR functional) NEAR/3 (neuroimag* OR image OR images OR imaging* OR scan OR scans OR mri OR tomograph*)):ti,ab,kw	107 808
#13	'functional cerebral localization*':ti,ab,kw OR fmri:ti,ab,kw OR magnetoencephalogra*:ti,ab,kw OR 'magneto encephalogra*':ti,ab,kw OR 'magneto-encephalogra*':ti,ab,kw OR meg$:ti,ab,kw	112 118
#14	(brain* NEAR/1 (activ* OR connectiv* OR correlate* OR function* OR image OR images OR imaging* OR mapping* OR network$ OR resonance* OR respons* OR tomograph*)):ti,ab,kw	158 861
#15	#5 OR #6 OR #7 OR #8 OR #9 OR #10 OR #11 OR #12 OR #13 OR #14	379 143
#16	#4 AND #15	7 392
#17	'in vitro study'/exp	7 399 088
#18	'in vitro':ti,ab,kw	1 824 797
#19	#17 OR #18	7 858 878
#20	#16 NOT #19	6 756
#21	#20 NOT ([animals]/lim NOT [humans]/lim)	6 542
#22	#21 AND [english]/lim AND [1990-2023]/py	6 349
#23	#22 NOT ('chapter'/it OR 'conference abstract'/it OR 'conference review'/it)	5 428
#24	#23 AND ('review'/it OR review:ti OR 'meta analysis':ti OR metaanalysis:ti)	1 341
#25	#23 NOT #24	4087

Interface: embase.com

Table S3. Search Terms: Web of Science

No	Terms	Results
1	TI=((Autism* or autist* or asperger* or "Kanner* Syndrome*")) OR AB=((Autism* or autist* or asperger* or "Kanner* Syndrome*")) OR AK=((Autism* or autist* or asperger* or "Kanner* Syndrome*"))	89 317
2	TI=((pervasive NEAR/2 (development* or disorder*))) OR AB=((pervasive NEAR/2 (development* or disorder*))) OR AK=((pervasive NEAR/2 (development* or disorder*)))	3 493
3	#1 OR #2	90 674
4	TI=(((echoplanar* or "echo-planar*" or "echo planar*" or functional) NEAR/2 (neuroimag* or image or images or imaging* or scan or scans or mri or tomograph*))) OR AB=(((echoplanar* or "echo-planar*" or "echo planar*" or functional) NEAR/2 (neuroimag* or image or images or imaging* or scan or scans or mri or tomograph*))) OR AK=(((echoplanar* or "echo-planar*" or "echo planar*" or functional) NEAR/2 (neuroimag* or image or images or imaging* or scan or scans or mri or tomograph*)))	86 061
5	TI=(("functional cerebral localization*" or fMRI or magnetoencephalogra* or "magneto-encephalogra*" or "magneto encephalogra*" or MEG$)) OR AB=(("functional cerebral localization*" or fMRI or magnetoencephalogra* or "magneto-encephalogra*" or "magneto encephalogra*" or MEG$)) OR AK=(("functional cerebral localization*" or fMRI or magnetoencephalogra* or "magneto-encephalogra*" or "magneto encephalogra*" or MEG$))	104 399
6	TI=((brain* NEAR/1 (activ* or connectiv* or correlate* or function* or image or images or imaging* or mapping* or network$ or resonance* or respons* or tomograph* or radiograph*))) OR AB=((brain* NEAR/1 (activ* or connectiv* or correlate* or function* or image or images or imaging* or mapping* or network$ or resonance* or respons* or tomograph* or radiograph*))) OR AK=((brain* NEAR/1 (activ* or connectiv* or correlate* or function* or image or images or imaging* or mapping* or network$ or resonance* or respons* or tomograph* or radiograph*)))	174 772
7	#4 OR #5 OR #6	286 492
8	#3 AND #7	4 672
9	(#8) NOT TS=("in vitro")	4 634
10	#9 AND LA=(English) Timespan: 1990-01-01 to 2023-12-31	4 542
11	#10 NOT (DT==("MEETING ABSTRACT" OR "BOOK CHAPTER" OR "LETTER" OR "BOOK REVIEW" OR "NEWS ITEM")) Timespan: 1990-01-01 to 2023-12-31	4 356
12	#11 AND TI=(review OR 'meta analysis' OR metaanalysis)	207
13	#11 NOT #12	4 149

Interface: Clarivate Analytics.

Table S4. Search Terms: PsycInfo

No	Terms	Results
S01	DE "Autism Spectrum Disorders" OR DE "Autistic Traits"	56 700
S02	TI ((Autism* or autist* or asperger* or (Kanner* N2 Syndrome*))) OR AB ((Autism* or autist* or asperger* or (Kanner* N2 Syndrome*))) OR KW ((Autism* or autist* or asperger* or (Kanner* N2 Syndrome*)))	68 095
S03	TI ((pervasive N2 (development* or disorder*))) OR AB ((pervasive N2 (development* or disorder*))) OR KW ((pervasive N2 (development* or disorder*)))	3 634
S04	S1 OR S2 OR S3	69 887
S05	DE "Functional Magnetic Resonance Imaging"	29 579
S06	DE "Stereotaxic Atlas"	4 751
S07	DE "Magnetoencephalography"	5 268
S08	DE "Magnetic Resonance Imaging"	69 570
S09	TI functional* OR AB functional* OR KW functional*	242 716
S10	S8 AND S9	27 743
S11	TI (((echoplanar* or "echo planar*" or functional) N2 (neuroimag* or image or images or imaging* or scan or scans or mri or tomograph*))) OR AB (((echoplanar* or "echo planar*" or functional) N2 (neuroimag* or image or images or imaging* or scan or scans or mri or tomograph*))) OR KW (((echoplanar* or "echo planar*" or functional) N2 (neuroimag* or image or images or imaging* or scan or scans or mri or tomograph*)))	43 618
S12	TI (("functional cerebral localization*" or fMRI or magnetoencephalogra* or magneto-encephalogra* or "magneto encephalogra*" or MEG#)) OR AB (("functional cerebral localization*" or fMRI or magnetoencephalogra* or magneto-encephalogra* or "magneto encephalogra*" or MEG?)) OR KW (("functional cerebral localization*" or fMRI or magnetoencephalogra* or magneto-encephalogra* or "magneto encephalogra*" or MEG#))	45 391
S13	TI ((brain* N1 (activ* or connectiv* or correlate* or function* or image or images or imaging* or mapping* or network# or resonance* or respons* or tomograph* or radiograph*))) OR AB ((brain* N1 (activ* or connectiv* or correlate* or function* or image or images or imaging* or mapping* or network# or resonance* or respons* or tomograph* or radiograph*))) OR KW ((brain* N1 (activ* or connectiv* or correlate* or function* or image or images or imaging* or mapping* or network# or resonance* or respons* or tomograph* or radiograph*)))	77 665
S14	S5 OR S6 OR S7 OR S10 OR S11 OR S12 OR S13	124 240
S15	S4 AND S14	3 198
S16	S15 AND Narrow by Language: - english	3 198
S17	S16 AND Limiters - Publication Year: 1990-2024	3 151

S18	S17 AND Limiters - Publication Year: 1990-2024; Publication Type: All Books, Encyclopedia; Document Type: Chapter, Editorial, Encyclopedia Entry, Interview, Letter, Obituary, Poetry, Review-Book, Review-Media, Review-Software & Other	189
S19	S17 NOT S18	2 899
S20	((DE "Systematic Review" OR DE "Meta Analysis" OR DE "Literature Review")) OR TI ((review OR "meta analysis" OR metaanalysis))	219 231
S21	S19 AND S20	136
S22	S19 NOT S20	2 763

Interface: EBSCO.

Table S5. Demographic and Quality Metrics

Author	Demographics					Quality assessment[d]						Sensory modality	Reference space	Coordinates	Notes[i]
	Group[a]	n	Gender[b]		Age[c]	ADOS and/or ADI-R	Group difference			Corrected cluster threshold p < .05[g]	Whole-brain coverage (mm)[h]				
			F	M			Age[e]	IQ[e]	Motion[f]						
Balsters et al. (1)	ASC	16	0	16	21	✓	✓	✓	✓	✓	✓	visual	MNI	Text	
	NT	20	0	20	22										
Björnsdotter et al. (2)	ASC	37	10	27	11	✓	✓	NT > ASC [male]	ASC > NT [female]; disc.; cov.	✓	✓	visual	Tal	Table 2	Masking; [Replication cohort]; Measures for combined gender groups not specified
	NT	37[j]	13	24	12										
Bolling et al. (3)	ASC	21	6	15	13	✓	✓	✓	✓	h: p < 0.05 k: 20	✓	visual	Tal	Table 4	
	NT	19	5	14	13										
Caria et al. (4)	ASC	8	2	6	23	✓	✓	-	covariate	✓	✓	auditory	MNI	Table 5	IQ not specified
	NT	14	8	6	24										
D'Cruz et al. (5)	ASC	17	5	12	17	✓	✓	NT > ASC [verbal]	motion correction	✓	FOV: 200^2; Slice number: 15; Slice thickness: 5; Slice gap: 1	visual	MNI	Table 3	'FOV typically extending from the dorsal neocortex to dorsal pons'; IQ measure not specified
	NT	23	5	18	19										
Fan et al. (6)	ASC	12	3	9	30	✓	✓	✓	discarded; covariate	h: p < 0.01 and e: p < 0.05	✓	visual	MNI	Table 2	
	NT	12	2	10	28										
Freitag et al. (7)	ASC	14	2	13	18	✓	✓	✓	discarded; covariate	h: p < 0.001 k: 20	FOV: -; Slice number: 36; Slice thickness: -; Slice gap: -	visual	MNI	Table 3	Masking
	NT	14	2	13	19										
Gomot et al. (8)	ASC	12	0	12	14	✓	✓	✓	motion correction	h: p < 0.001	✓	auditory	Tal	Table 2	Watched unrelated video during paradigm
	NT	12	0	12	14										
Gomot et al. (9)	ASC	-	-	-	-	✓	✓	✓	motion correction	h: p < 0.001	✓	auditory	Tal	Table 3	Same sample as in (8) (confirmed through personal correspondence)
	NT	-	-	-	-										
Groen et al. (10)	ASC	16	4	12	15	✓	✓	✓	discarded; covariate	✓	FOV: 224^2; Slice number: 31; Slice thickness: -; Slice gap: 0.5	auditory	MNI	Table 5	'[S]mall part of the superior parietal cortex was not scanned in a number of participants'
	NT	26	5	21	16										

Hames et al. (11)	ASC	6	2	4	17	✓	-	-	motion correction	✓	✓	visual	MNI	Table 2	Standard deviation of age not specified; IQ not specified; Fixed-effects analysis
	NT	6	2	4	16										
Jack et al. (12)	ASC	45	45	0	13	✓	✓	✓	✓	✓	✓	visual	MNI	Table 2	Multi-site study; Split clusters; [Matched female sample]
	NT	45	45	0	13										
Kinard et al. (13)	ASC	22	3	19	15	✓	✓	NT > ASC [verbal]	✓	✓	✓	visual	MNI	Table S3	
	NT	20	11	9	15										
Libero et al. (14)	ASC	21	4	17	26	-	✓	✓	✓	✓	✓	visual	MNI	Table 3	'[FOV] covered most of the brain'
	NT	22	5	17	25										
Sapey-Triomphe et al. (15)	ASC	16	0	16	14	-	✓	NT > ASC	discarded; covariate	✓	✓	visual	MNI	Table S5	IQ measure not specified
	NT	19	0	19	14										
Sapey-Triomphe et al. (16)	ASC	25	13	12	32	-	✓	✓	discarded; covariate	✓	✓	visual; auditory	MNI	Table S4.2	
	NT	26	13	13	31										
Shafritz et al. (17)	ASC	15	2	13	22	✓	✓	✓	discarded	h: p < .001 k: 10	✓	visual	Tal	Text; Table 1	
	NT	14	2	12	24										
Sharer et al. (18)	ASC	17	2	15	11	✓	✓	NT > ASC	discarded	✓	FOV: -; Slice number: -; Slice thickness: -; Slice gap: -	visual	MNI	Table2	
	NT	36	8	28	11										
Yang et al. (19)	ASC	31	0	31	11	✓	✓	✓	✓	✓	✓	visual	MNI	Table 3	Masking; [Full sample]
	NT	17	0	17	11										

[a]Participants after exclusions and/or within the relevant subset, to the extent specified. [b]For studies where the gender of excluded participants was not specified, female and male total may not correspond to sample total. [c]If age reported for each gender separately, weighted group average used. Age reported in months converted to years. Years rounded to the closest integer. [d]'-', not carried out, not reported, or insufficient information to assess. [e]Where not tested, heuristically considered no group difference if 'matched' on variable, or if both standard deviations overlapped both means. Subscales are referenced to the extent that significant differences on them were reported. [f]'Discarded', criterion for exclusion of volumes and/or participants. 'Covariate', motion as a regressor of no interest. [g]'h', voxel height. 'k'/'e', cluster extent. [h]Considered whole-brain coverage if explicitly stated so, or if width, length, height dimensions exceeded 140 x 167 x 93 mm (20). [i]'Masking', inference space restricted during analysis. For example, only the joined set of voxels activated within either group considered. [j]Information here assumes a typo in the referenced study on from which group one participant was excluded to achieve equal sample sizes. ASC, autism spectrum condition; NT, neurotypically developing; F, female; M, male; ADOS, Autism

Diagnostic Observation Schedule; ADI-R, Autism Diagnostic Interview-Revised; IQ, intelligence quotient; MNI, Montreal Neurological Institute; Tal, Talairach; FOV, field of view.

Table S6. Group Differences on Behavioral Measures

Author	Task	Performance outcome	Test	Difference[a]
Balsters et al. (1)	Report if outcome was expected or unexpected, for various agents	[Overall] Accuracy	F	NT > ASC
		[Expected] Accuracy	F	NT > ASC
		[Unexpected] Accuracy	F	NT > ASC
Björnsdotter et al. (2)	No task	-	-	-
Bolling et al. (3)	Throw shape to co-players	No relevant	-	-
Caria et al. (4)	No task	-	-	-
D'Cruz et al. (5)	Two-choice set-shifting task	Perseverance error	F	-
		Failure to maintain set	F	-
	Four-choice set-shifting task	Perseverance error	F	-
		Failure to maintain set	F	-
Fan et al. (6)	Report direction of target	[Overall] Accuracy	T	NT > ASC
		[Overall] Reaction time	T	-
		[Validity] Reaction time difference	U	-
		[Validity] Error difference	T	-
		[Flanker conflict] Reaction time difference	U	-
		[Flanker conflict] Error difference	T	ASC > NT

Freitag et al. (7)	Report if biological or scrambled motion	[Overall] Error rate	T	-
		[Biological] Reaction time	T	NT > ASC
		[Scrambled] Reaction time	T	NT > ASC
Gomot et al. (8)	No task	-	-	-
Gomot et al. (9)	Report novel sounds	Reaction time	T	ASC > NT
		True positives	-	-
		False positives	-	-
Groen et al. (10)	Report pseudowords	No relevant	-	-
Hames et al. (11)	Report direction of target	[Flanker conflict] Accuracy	F	-
		[Flanker conflict] Reaction time	F	-
Jack et al. (12)	No task	-	-	-
Kinard et al. (13)	Report if reward or non-reward is likely	[Overall] Accuracy	W	NT > ASC
		[Overall] Reaction time	T	NT > ASC
		[Social condition] Accuracy	W	NT > ASC
		[Social condition] Reaction time	T	NT > ASC
		[Non-social condition] Accuracy	W	-
		[Non-social condition] Reaction time	T	NT > ASC
Libero et al. (14)	Report whether ordinary or unusual[b]	Accuracy	F	NT > ASC
		Reaction time	F	-
Sapey-Triomphe et al. (15)	Report when a (meaningful or meaningless) pattern emerges[c]	[All conditions] Accuracy	F	-
		[All conditions] Reaction time	F	-

Sapey-Triomphe et al. (16)	Report prediction and perception of rotation direction	[Prediction] Accuracy	T	-
		[Unambiguous] Accuracy	T	-
		[Ambiguous] According to contingency	T	-
Shafritz et al. (17)	Report target, novel, and standard shapes	[Overall] Accuracy	F	NT > ASC
		[Overall] Reaction time	F	-
		[Target] Accuracy	T	NT > ASC
		[Target] Reaction time	T	-
		[Novel] Accuracy	T	-
		[Novel] Reaction time	T	-
		[Standard] Accuracy	T	-
		[Standard] Reaction time	T	-
Sharer et al. (18)	Press button corresponding to target location	[Overall] Accuracy	T	-
		[Run 1-3] [Sequence] Reaction time	F	-
		[Run 1] [Sequence] Reaction time	F	-
		[Run 2] [Sequence] Reaction time	F	-
		[Run 3] [Sequence] Reaction time	F	-
Yang et al. (19)	No task	-	-	-

[a]Reaction time: [group 1] > [group 2] indicates that [group 1] was faster. ASC, Autism Spectrum Condition; NT, neurotypically developing. [b]Ordinary/unusual was not used as a factor in the model. [c]Additional conditions were also included in the model.

Table S7. Non-Significant Between-Group Experiments

Author	Group size	Paradigm	Experiment	Prediction
Alaerts et al. (21)	15 ASC; 15 NT	Elements represent human motion, or scrambled motion	intact > scrambled motion	formation

Bathelt et al. (22)	50 ASC; 49 NT	Mooney images	face > non-face [Correct trials]	formation
Catarino et al. (23)	12 ASC; 12 NT	Last word of a statement does or does not violate semantic knowledge	incongruent > congruent [Concrete sentences]	violation
			incongruent > congruent [Emotional sentences]	violation
Charpentier et al. (24)	14 ASC; 16 NT	Repetitions of standard, novel, and deviant sounds	deviants > standard [Neutral and emotional]	violation
			novels > standard [Neutral and emotional]	violation
D'Cruz et al. (5)	17 ASC; 23 NT	Set-shifting task	first reversal > first and following expected [Two-choice task]	violation
Dunham et al. (25)	18 ASC; 30 NT	Video and audio of speaker is synchronous or asynchronous	asynchronous > synchronous speech	violation
Groen et al. (10)	16 ASC; 26 NT	Contents of a statement is or is not congruent with expectations with regards to voice	speaker incongruent > congruent	violation
		Last word of a statement does or does not violate semantic knowledge	anomaly > no anomaly [Semantic knowledge]	violation
Herrington et al. (26)	10 ASC; 10 NT	Elements represent human motion, or scrambled motion	walker > random walker	formation
Jack et al. (12)	47 ASC; 47 NT	Elements represent human motion, or scrambled motion	biological > scrambled motion [Matched male sample]	formation
Kinard et al. (13)	22 ASC; 20 NT	Cues probabilistically signal upcoming reward or non-reward	signed prediction error [Social condition]	violation
				violation

			thresholded unsigned prediction error [Non-social condition]	violation
			signed prediction error [Non-social condition]	
Marsh and Hamilton (27)	18 ASC; 19 NT	Agent reaches for an object in a rational/irrational way	irrational > rational [Possible and impossible]	violation
Mosner et al. (28)	16 ASC; 14 NT	Cues probabilistically signal upcoming reward or non-reward	thresholded unsigned prediction error	violation
			signed prediction error	violation
Randeniya et al. (29)	21 ASC; 21 NT	Predict location of a coin throw, based on wide or narrow prior and likelihood	wide >/< narrow prior	formation
			wide >/< narrow likelihood	formation
Rosenblau et al. (30)	20 ASC; 26 NT	Predict peers' preference for various items	[Model-free] prediction errors	violation
Sapey-Triomphe et al. (16)	25 ASC; 26 NT	Alternating tones probabilistically signals upcoming (ambiguous or unambiguous) rotation direction	prior mean [Third-level]	formation
			prior mean [Second-level]	formation
			prior precision [Third-level]	formation
			prior precision [Second-level]	formation
			precision-weighted prediction error [Third-level]	violation
			absolute perceptual prediction error	violation

Sharer et al. (18)	17 ASC; 36 NT	Target locations follow a set or a random sequence	sequence > random [Run 2]	formation
			sequence > random [Run 3]	formation
Stickel et al. (31)	18 ASC; 17 NT	Combinations of pleasant or unpleasant images/sounds or images/odors	incongruent > congruent [Auditory-visual condition]	violation
			incongruent > congruent [Olfactory-visual condition]	violation
Tesink et al. (32)	24 ASC; 24 NT	Semantic content congruent or not with speaker's age, gender, or social background	speaker incongruent > congruent	violation
Tietze et al. (33)	16 ASC; 16 NT	Speakers' lip movements fit or do not fit the audible words	audiovisual incongruency > congruency	violation
Utzerath et al. (34)	22 ASC; 22 NT	Expected or unexpected repetitions/alternations of objects	unexpected > expected	violation
Utzerath et al. (35)	[same sample as (34)]	Kanizsa illusion	illusory contour > no illusory contour	formation
Valles-Capetillo et al.[b] (36)	26 ASC; 15 NT	Last part of sentence congruent or not with rest of sentence (or last sentence with preceding sentences)	incongruent > congruent [One sentence]	violation
	25 ASC; 15 NT [same sample]		incongruent > congruent [Multisentence]	violation

[a]Participants after exclusions and/or within the relevant subset, to the extent specified. [b]Information on experiments from personal correspondence. ASC, Autism Spectrum Condition; NT, neurotypically developing.

Table S8. NT Within-Group Experiments

Author	Group size[a]	Paradigm	Experiment	Prediction
Bolling et al. (3)	19 NT	Co-players follow or break the rules of a game	rule violation > fair play [Cybershape]	violation
Caria et al. (4)	14 NT	Music or random sequence of tones	[Happy] music > control	formation

			[Standard and favorite]	
			[Sad] music > control [Standard and favorite]	formation
Catarino et al. (23)	12 NT	Last word of a statement does or does not violate semantic knowledge	incongruent > congruent [Concrete sentences]	violation
D'Cruz et al. (5)	23 NT	Set-shifting task	first reversal > first and following expected [Two-choice task]	violation
			first reversal > first and following expected [Four-choice task]	violation
Fan et al. (6)	12 NT	Flankers congruent or incongruent with target	incongruent > congruent	violation
Freitag et al. (7)	14 NT	Elements represent human motion, or scrambled motion	biological > scrambled motion	formation
Groen et al. (10)	26 NT	Contents of a statement is or is not congruent with expectations with regards to voice	speaker incongruent > congruent	violation
		Last word of a statement does or does not violate real-world knowledge	anomaly > no anomaly [World-knowledge]	violation
		Last word of a statement does or does not violate semantic knowledge	anomaly > no anomaly [Semantic knowledge]	violation
		Normal spoken sentences, or speech-like noise fragments	normal sentence > speech-like noise	formation
Jack et al. (12)	45 NT	Elements represent human motion, or scrambled motion	biological > scrambled motion [Matched female sample]	formation

	47 NT		biological > scrambled motion [Matched male sample]	formation
Kinard et al. (13)	20 NT	Cues probabilistically signal upcoming reward or non-reward	signed prediction error [Social condition]	violation
Libero et al. (14)	22 NT	Agent uses an object in an ordinary or unusual way	unusual > ordinary	violation
Marsh and Hamilton (27)	19 NT	Agent reaches for an object in a rational/irrational way	irrational > rational [Possible and impossible]	violation
Rosenblau et al. (30)	26 NT	Predict peers' preference for various items	[Model-free] prediction errors	violation
Sapey-Triomphe et al. (15)	19 NT	Elements move to form recognizable or non-recognizable objects	meaningful > meaningless [Contour and texture]	formation
Sapey-Triomphe et al. (16)	26 NT	Alternating tones probabilistically signals upcoming (ambiguous or unambiguous) rotation direction	prior mean [Third-level]	formation
			prior mean [Second-level]	formation
			prior precision [Third-level]	formation
			precision-weighted prediction error [Second-level]	violation
			absolute perceptual prediction error	violation
Sharer et al. (18)	36 NT	Target locations follow a set or a random sequence	sequence > random [Runs 1-3]	formation

Valles-Capetillo et al.[a] (36)	15 NT	Last part of sentence congruent or not with rest of sentence (or last sentence with preceding sentences)	incongruent > congruent [Multisentence]	violation

[a]Information on experiments from personal correspondence. NT, neurotypically developing.

Table S9. NT Within-Group Results

Area	Volume (mm^3)	ALE	Z	p	MNI x	MNI y	MNI z
Supramarginal gyrus	784	0.019	4.33	< .00005	-44	-44	40
Inferior parietal lobule		0.014	3.56	< .0005	-40	-34	44
Claustrum	768	0.017	4.01	< .00005	-34	20	-4

NT, neurotypically developing; MNI, Montreal Neurological Institute; ALE, activation likelihood estimation.

Supplementary Figures

Figure S1. NT Within-Group Results

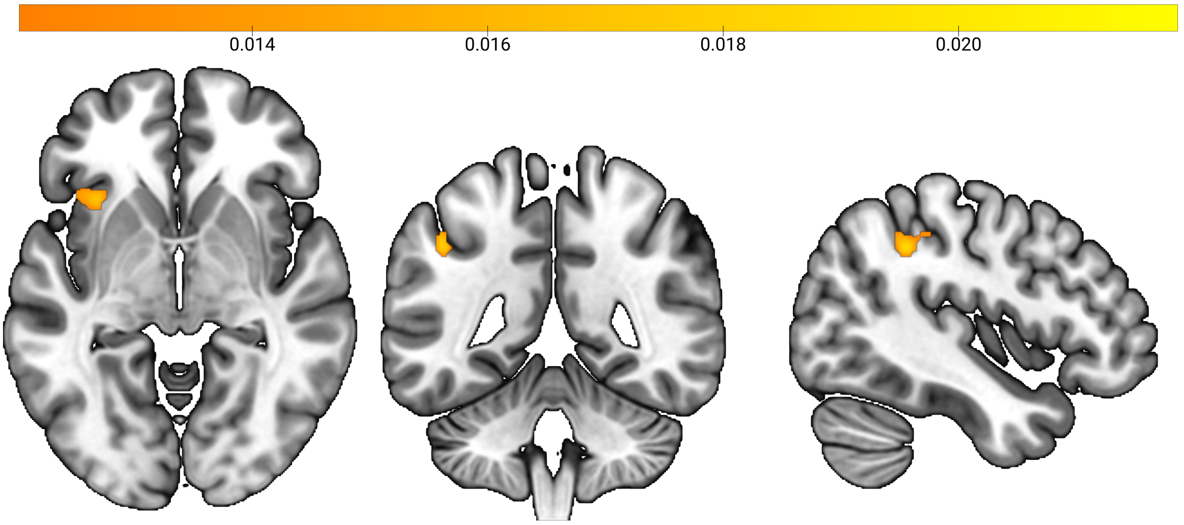

Slice displayed: x = -44.05, y = -45.86, z = -3.91. Legend: ALE values. NT, neurotypically developing. ALE, activation likelihood estimation.

References

1. Balsters JH, Apps MA, Bolis D, Lehner R, Gallagher L, Wenderoth N. Disrupted prediction errors index social deficits in autism spectrum disorder. Brain. 2017;140(1):235-46. doi: 10.1093/brain/aww287. PubMed PMID: 28031223; PubMed Central PMCID: PMC5379861.
2. Bjornsdotter M, Wang N, Pelphrey K, Kaiser MD. Evaluation of Quantified Social Perception Circuit Activity as a Neurobiological Marker of Autism Spectrum Disorder. JAMA Psychiatry. 2016;73(6):614-21. doi: 10.1001/jamapsychiatry.2016.0219. PubMed PMID: 27096285; PubMed Central PMCID: PMC6475601.
3. Bolling DZ, Pitskel NB, Deen B, Crowley MJ, McPartland JC, Kaiser MD, et al. Enhanced neural responses to rule violation in children with autism: a comparison to social exclusion. Dev Cogn Neurosci. 2011;1(3):280-94. doi: 10.1016/j.dcn.2011.02.002. PubMed PMID: 21743819; PubMed Central PMCID: PMC3129780.

4. Caria A, Venuti P, de Falco S. Functional and dysfunctional brain circuits underlying emotional processing of music in autism spectrum disorders. Cereb Cortex. 2011;21(12):2838-49. Epub 20110428. doi: 10.1093/cercor/bhr084. PubMed PMID: 21527791.
5. D'Cruz AM, Mosconi MW, Ragozzino ME, Cook EH, Sweeney JA. Alterations in the functional neural circuitry supporting flexible choice behavior in autism spectrum disorders. Transl Psychiatry. 2016;6(10):e916. Epub 20161011. doi: 10.1038/tp.2016.161. PubMed PMID: 27727243; PubMed Central PMCID: PMC5315543.
6. Fan J, Bernardi S, Van Dam NT, Anagnostou E, Gu X, Martin L, et al. Functional deficits of the attentional networks in autism. Brain Behav. 2012;2(5):647-60. Epub 20120827. doi: 10.1002/brb3.90. PubMed PMID: 23139910; PubMed Central PMCID: PMC3489817.
7. Freitag CM, Konrad C, Haberlen M, Kleser C, von Gontard A, Reith W, et al. Perception of biological motion in autism spectrum disorders. Neuropsychologia. 2008;46(5):1480-94. Epub 20080105. doi: 10.1016/j.neuropsychologia.2007.12.025. PubMed PMID: 18262208.
8. Gomot M, Bernard FA, Davis MH, Belmonte MK, Ashwin C, Bullmore ET, et al. Change detection in children with autism: an auditory event-related fMRI study. Neuroimage. 2006;29(2):475-84. Epub 20050822. doi: 10.1016/j.neuroimage.2005.07.027. PubMed PMID: 16115783.
9. Gomot M, Belmonte MK, Bullmore ET, Bernard FA, Baron-Cohen S. Brain hyper-reactivity to auditory novel targets in children with high-functioning autism. Brain. 2008;131(Pt 9):2479-88. Epub 20080731. doi: 10.1093/brain/awn172. PubMed PMID: 18669482.
10. Groen WB, Tesink C, Petersson KM, van Berkum J, van der Gaag RJ, Hagoort P, et al. Semantic, factual, and social language comprehension in adolescents with autism: an FMRI study. Cereb Cortex. 2010;20(8):1937-45. Epub 20091216. doi: 10.1093/cercor/bhp264. PubMed PMID: 20016003.
11. Hames EC, Rajmohan R, Fang D, Anderson R, Baker M, Richman DM, et al. Attentional Networks in Adolescents with High-functioning Autism: An fMRI Investigation. Open Neuroimag J. 2016;10:102-10. Epub 20160930. doi: 10.2174/1874440001610010102. PubMed PMID: 27843514; PubMed Central PMCID: PMC5074002.
12. Jack A, Sullivan CAW, Aylward E, Bookheimer SY, Dapretto M, Gaab N, et al. A neurogenetic analysis of female autism. Brain. 2021;144(6):1911-26. doi: 10.1093/brain/awab064. PubMed PMID: 33860292; PubMed Central PMCID: PMC8320285.
13. Kinard JL, Mosner MG, Greene RK, Addicott M, Bizzell J, Petty C, et al. Neural Mechanisms of Social and Nonsocial Reward Prediction Errors in Adolescents with Autism Spectrum Disorder. Autism Res. 2020;13(5):715-28. Epub 20200211. doi: 10.1002/aur.2273. PubMed PMID: 32043748; PubMed Central PMCID: PMC8112190.
14. Libero LE, Maximo JO, Deshpande HD, Klinger LG, Klinger MR, Kana RK. The role of mirroring and mentalizing networks in mediating action intentions in autism. Mol Autism. 2014;5(1):50. Epub 20141014. doi: 10.1186/2040-2392-5-50. PubMed PMID: 25352976; PubMed Central PMCID: PMC4210608.
15. Sapey-Triomphe LA, Boets B, Van Eylen L, Noens I, Sunaert S, Steyaert J, et al. Ventral stream hierarchy underlying perceptual organization in adolescents with autism. Neuroimage Clin. 2020;25:102197. Epub 20200125. doi: 10.1016/j.nicl.2020.102197. PubMed PMID: 32014827; PubMed Central PMCID: PMC6997624.

16. Sapey-Triomphe LA, Pattyn L, Weilnhammer V, Sterzer P, Wagemans J. Neural correlates of hierarchical predictive processes in autistic adults. Nat Commun. 2023;14(1):3640. Epub 20230619. doi: 10.1038/s41467-023-38580-9. PubMed PMID: 37336874; PubMed Central PMCID: PMC10279690.
17. Shafritz KM, Dichter GS, Baranek GT, Belger A. The neural circuitry mediating shifts in behavioral response and cognitive set in autism. Biol Psychiatry. 2008;63(10):974-80. Epub 20071004. doi: 10.1016/j.biopsych.2007.06.028. PubMed PMID: 17916328; PubMed Central PMCID: PMC2599927.
18. Sharer E, Crocetti D, Muschelli J, Barber AD, Nebel MB, Caffo BS, et al. Neural Correlates of Visuomotor Learning in Autism. J Child Neurol. 2015;30(14):1877-86. Epub 20150908. doi: 10.1177/0883073815600869. PubMed PMID: 26350725; PubMed Central PMCID: PMC4941625.
19. Yang YJ, Sukhodolsky DG, Lei J, Dayan E, Pelphrey KA, Ventola P. Distinct neural bases of disruptive behavior and autism symptom severity in boys with autism spectrum disorder. J Neurodev Disord. 2017;9:1. Epub 20170117. doi: 10.1186/s11689-017-9183-z. PubMed PMID: 28115995; PubMed Central PMCID: PMC5240249.
20. Muller VI, Cieslik EC, Laird AR, Fox PT, Radua J, Mataix-Cols D, et al. Ten simple rules for neuroimaging meta-analysis. Neurosci Biobehav Rev. 2018;84:151-61. Epub 20171124. doi: 10.1016/j.neubiorev.2017.11.012. PubMed PMID: 29180258; PubMed Central PMCID: PMC5918306.
21. Alaerts K, Swinnen SP, Wenderoth N. Neural processing of biological motion in autism: An investigation of brain activity and effective connectivity. Sci Rep. 2017;7(1):5612. Epub 20170717. doi: 10.1038/s41598-017-05786-z. PubMed PMID: 28717158; PubMed Central PMCID: PMC5514051.
22. Bathelt J, Koolschijn PCM, Geurts HM. Atypically slow processing of faces and non-faces in older autistic adults. Autism. 2022;26(7):1737-51. Epub 20211228. doi: 10.1177/13623613211065297. PubMed PMID: 34961340; PubMed Central PMCID: PMC9483195.
23. Catarino A, Luke L, Waldman S, Andrade A, Fletcher PC, Ring H. An fMRI investigation of detection of semantic incongruities in autistic spectrum conditions. Eur J Neurosci. 2011;33(3):558-67. Epub 20101229. doi: 10.1111/j.1460-9568.2010.07503.x. PubMed PMID: 21198976.
24. Charpentier J, Latinus M, Andersson F, Saby A, Cottier JP, Bonnet-Brilhault F, et al. Brain correlates of emotional prosodic change detection in autism spectrum disorder. Neuroimage Clin. 2020;28:102512. Epub 20201127. doi: 10.1016/j.nicl.2020.102512. PubMed PMID: 33395999; PubMed Central PMCID: PMC8481911.
25. Dunham K, Zoltowski A, Feldman JI, Davis S, Rogers B, Failla MD, et al. Neural Correlates of Audiovisual Speech Processing in Autistic and Non-Autistic Youth. Multisens Res. 2023;36(3):263-88. Epub 20230119. doi: 10.1163/22134808-bja10093. PubMed PMID: 36731524; PubMed Central PMCID: PMC10121891.
26. Herrington JD, Baron-Cohen S, Wheelwright SJ, Singh KD, Bullmore ET, Brammer M, et al. The role of MT+/V5 during biological motion perception in Asperger Syndrome: An fMRI study. Research in Autism Spectrum Disorders. 2007;1(1):14-27. doi: 10.1016/j.rasd.2006.07.002.

27. Marsh LE, Hamilton AF. Dissociation of mirroring and mentalising systems in autism. Neuroimage. 2011;56(3):1511-9. Epub 20110217. doi: 10.1016/j.neuroimage.2011.02.003. PubMed PMID: 21310248.
28. Mosner MG, McLaurin RE, Kinard JL, Hakimi S, Parelman J, Shah JS, et al. Neural Mechanisms of Reward Prediction Error in Autism Spectrum Disorder. Autism Res Treat. 2019;2019:5469191. Epub 20190701. doi: 10.1155/2019/5469191. PubMed PMID: 31354993; PubMed Central PMCID: PMC6634058.
29. Randeniya R, Vilares I, Mattingley JB, Garrido MI. Increased functional activity, bottom-up and intrinsic effective connectivity in autism. Neuroimage Clin. 2023;37:103293. Epub 20221213. doi: 10.1016/j.nicl.2022.103293. PubMed PMID: 36527995; PubMed Central PMCID: PMC9791168.
30. Rosenblau G, Korn CW, Dutton A, Lee D, Pelphrey KA. Neurocognitive Mechanisms of Social Inferences in Typical and Autistic Adolescents. Biol Psychiatry Cogn Neurosci Neuroimaging. 2021;6(8):782-91. Epub 20200715. doi: 10.1016/j.bpsc.2020.07.002. PubMed PMID: 32952091.
31. Stickel S, Weismann P, Kellermann T, Regenbogen C, Habel U, Freiherr J, et al. Audio-visual and olfactory-visual integration in healthy participants and subjects with autism spectrum disorder. Hum Brain Mapp. 2019;40(15):4470-86. Epub 20190713. doi: 10.1002/hbm.24715. PubMed PMID: 31301203; PubMed Central PMCID: PMC6865810.
32. Tesink CM, Buitelaar JK, Petersson KM, van der Gaag RJ, Kan CC, Tendolkar I, et al. Neural correlates of pragmatic language comprehension in autism spectrum disorders. Brain. 2009;132(Pt 7):1941-52. Epub 20090507. doi: 10.1093/brain/awp103. PubMed PMID: 19423680.
33. Tietze FA, Hundertmark L, Roy M, Zerr M, Sinke C, Wiswede D, et al. Auditory Deficits in Audiovisual Speech Perception in Adult Asperger's Syndrome: fMRI Study. Front Psychol. 2019;10:2286. Epub 20191010. doi: 10.3389/fpsyg.2019.02286. PubMed PMID: 31649597; PubMed Central PMCID: PMC6795762.
34. Utzerath C, Schmits IC, Buitelaar J, de Lange FP. Adolescents with autism show typical fMRI repetition suppression, but atypical surprise response. Cortex. 2018;109:25-34. Epub 20180908. doi: 10.1016/j.cortex.2018.08.019. PubMed PMID: 30286304.
35. Utzerath C, Schmits IC, Kok P, Buitelaar J, de Lange FP. No evidence for altered up- and downregulation of brain activity in visual cortex during illusory shape perception in autism. Cortex. 2019;117:247-56. Epub 20190326. doi: 10.1016/j.cortex.2019.03.011. PubMed PMID: 31005025.
36. Valles-Capetillo E, Kurtz MR, Kana RK. The Role of the Brain's Pragmatic Language Network in Reading Comprehension in Autistic Children. Autism Res. 2025;18(8):1550-62. Epub 20250623. doi: 10.1002/aur.70076. PubMed PMID: 40546092; PubMed Central PMCID: PMC12268268.
